# Intake of Fish and Marine n-3 Polyunsaturated Fatty Acids and Risk of Cardiovascular Disease Mortality: A Meta-Analysis of Prospective Cohort Studies

**DOI:** 10.3390/nu13072342

**Published:** 2021-07-09

**Authors:** Lan Jiang, Jinyu Wang, Ke Xiong, Lei Xu, Bo Zhang, Aiguo Ma

**Affiliations:** Institute of Nutrition and Health, School of Public Health, Qingdao University, Qingdao 266071, China; jiangl59@163.com (L.J.); wangjinyu@qdu.edu.cn (J.W.); kexiong@qdu.edu.cn (K.X.); xulei951121@163.com (L.X.); zhangzhang19940516@163.com (B.Z.)

**Keywords:** fish, n-3 polyunsaturated fatty acid, cardiovascular disease mortality, meta-analysis, prospective cohort studies

## Abstract

Previous epidemiological studies have investigated the association of fish and marine n-3 polyunsaturated fatty acids (n-3 PUFA) consumption with cardiovascular disease (CVD) mortality risk. However, the results were inconsistent. The purpose of this meta-analysis is to quantitatively evaluate the association between marine n-3 PUFA, fish and CVD mortality risk with prospective cohort studies. A systematic search was performed on PubMed, Web of Science, Embase and MEDLINE databases from the establishment of the database to May 2021. A total of 25 cohort studies were included with 2,027,512 participants and 103,734 CVD deaths. The results indicated that the fish consumption was inversely associated with the CVD mortality risk [relevant risk (RR) = 0.91; 95% confidence intervals (CI) 0.85−0.98]. The higher marine n-3 PUFA intake was associated with the reduced risk of CVD mortality (RR = 0.87; 95% CI: 0.85–0.89). Dose-response analysis suggested that the risk of CVD mortality was decreased by 4% with an increase of 20 g of fish intake (RR = 0.96; 95% CI: 0.94–0.99) or 80 milligrams of marine n-3 PUFA intake (RR = 0.96; 95% CI: 0.94–0.98) per day. The current work provides evidence that the intake of fish and marine n-3 PUFA are inversely associated with the risk of CVD mortality.

## 1. Introduction

Cardiovascular diseases (CVD) are a group of disorders of the heart and blood vessels, including coronary heart disease, cerebrovascular disease, rheumatic heart disease and other conditions. The global CVD mortality increased 12.5% from 2005 to 2015. 17.9 million people died of CVD in 2015 [1]. In addition to drug treatment, the potential role of dietary components hasreceived increased attention. Previous studies have shown the effectiveness of healthy dietary patterns and components for the prevention of CVD and other diseases [2,3,4]. Fish is rich in various nutrients (e.g., protein, vitamin D and polyunsaturated fatty acids) and may have a beneficial role in preventing CVD events [5,6].

Marine n-3 polyunsaturated fatty acids (n-3 PUFA)—including eicosapentaenoic acid (EPA), docosahexaenoic acid (DHA) and docosapentaenoic acid (DPA)—mainly exist in fatty fish. A high consumption of n-3 PUFA from fatty fish led to an increase in high-density lipoprotein and a decrease in inflammation factors [7,8]. Besides, n-3 PUFA may improve heart rate and blood pressure through improving left ventricular diastolic filling or augmenting vagal tone [9].

Previous epidemiological studies have investigated the association of fish consumption with CVD mortality risk [10,11]. A recent meta-analysis of prospective observational studies revealed a negative association between fish intake and CVD mortality risk [12]. In recent years, another 11 prospective cohort studies investigated the association between fish intake and CVD mortality risk, but the findings were inconsistent [13,14,15,16]. The EPIC-Netherlands cohort study suggested that fish was not associated with the risk of CVD mortality [17]. In contrast, the NIH-AARP Diet and Health Study found that fish had a protective effect on CVD mortality risk [18]. To our knowledge, there has been no meta-analysis of prospective observational studies for investigating the association of marine n-3 PUFA consumption with CVD mortality risk. Therefore, we conducted this meta-analysis to comprehensively investigate the associations between fish, marine n-3 PUFA intake and CVD mortality risk. Furthermore, dose-response analyses were conducted to quantify the associations.

## 2. Materials and Methods

### 2.1. Data Sources and Search Strategy

Systematic search was performed on PubMed, Web of Science, Embase and MEDLINE from the establishment to May 2021. The search was limited to English literature, and the search keywords were “fish”, “seafood”, “fish products”, “fish oil”, “EPA”, “eicosapentaenoic acid”, “DHA”, “docosahexaenoic acid”, “DPA”, “docosapentaenoic acid”, “n-3 polyunsaturated fatty acid”, “ω-3 polyunsaturated fatty acid”, “n-3 PUFA”, “ω-3 PUFA”, “cardiovascular diseases”, “CVD”, “cardiovascular”, “cohort”, “follow-up”, “prospective” and “longitudinal”.

### 2.2. Study Selection

Two project members (L.J. and B.Z.) independently screened all titles and abstracts of the retrieved studies. Disagreements regarding the inclusion of the studies and the interpretation of the data were resolved by discussion among investigators. The studies were included in this meta-analysis if they met the following criteria: (1) study design: prospective cohort studies; (2) exposure: fish and marine n-3 PUFA; (3) source of n-3 PUFA: marine-derived n-3 PUFA (DHA, DPA, and EPA); and (4) outcomes: total CVD mortality which was reported as multivariate-adjusted relative risk (RR) and 95% confidence intervals (CI). The studies were excluded with the following criteria: (1) irrelevant; (2) not human studies; (3) not cohort studies; (4) not English studies.

### 2.3. Data Extraction

The following information was extracted from each eligible study: first author’s surname; the year of publication; country; age; follow-up duration; the number of CVD deaths, sample size; gender; exposure levels; multivariate-adjusted RR with 95% CI for the highest versus the lowest category of fish or marine n-3 PUFA intake; adjusted covariates. Consumption of fish and marine n-3 PUFA was collected with adjusted RR (95% CI) to conduct dose-response analyses. Newcastle–Ottawa Quality Assessment Scale (NOS) was adopted to evaluate the quality of each included study [19]. The NOS score ranges from 0 (bad) to 9 (good).

The quality evaluation was performed independently by two project members (L.J. and B.Z.). The NOS quality score system assessed 3 items: population selection, comparability of the groups and outcome assessment. Any discrepancies in grading the quality were addressed by group discussion.

### 2.4. Statistical Analyses

All statistical analyses were performed using Stata (Version 15.1). RRs with 95% CI for all the exposure categories were extracted for the analysis. The main effect was RRs with 95% CI. A two-tailed *p* < 0.05 was considered as statistically significant. The summary estimation was conducted through the comparison of the highest and the lowest category. Heterogeneity was assessed using the I^2^ statistic. In the case of heterogeneity for I^2^ > 50%, a random-effect model was adopted to pool the results. Otherwise, a fixed effect model was chosen.

Sensitivity analysis was implemented by deleting one study at a time. Subgroup analyses and meta-regression were performed to identify the possible sources of heterogeneity. In the subgroup analyses, the included studies were stratified by location (Asia, Europe plus America, Oceania and Five Continents), follow-up duration (<9 and ≥9 years), etc. In meta-regression, gender, country, dropout rate, follow-up duration, CVD history, adjustment for diabetes and adjustment for smoking were used as the covariates. Potential publication bias was accessed using funnel plots and Egger’s test (*p* < 0.1 was considered statistically significant).

Non-linear dose-response analyses were performed to evaluate the relationship between fish, marine n-3 PUFA intake and CVD mortality risk [20]. Potential non-linear correlation was accessed by modeling the consumption level using restricted cubic splines. The distribution of four fixed knots were 5%, 35%, 65% and 95% [21]. Owing to the discrepancy of fish and marine n-3 PUFA intake categories, we selected studies with clear doses to perform dose-response analyses. Among each study, we used the median or mean consumption of fish and marine n-3 PUFA from each category. For open-ended categories, we set the lower boundary to zero in lowest category and the width of the category to be the same as the adjacent interval in the highest one [12,22].

## 3. Results

### 3.1. Literature Search and Study Characteristics

**Figure 1:**
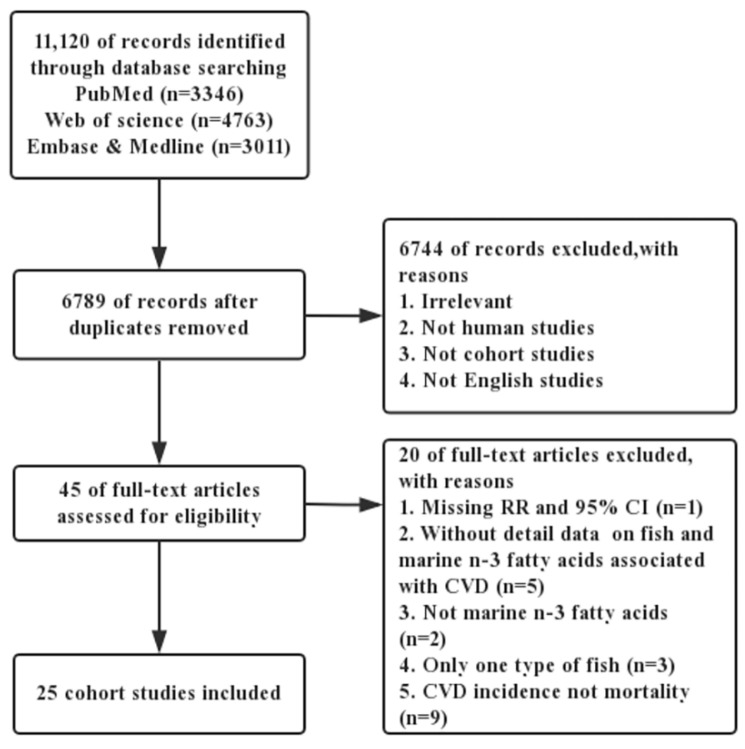
The flowchart for detailed steps of literature search.

The process of literature search is presented in Figure 1. A total of 11,120 articles were identified. After screening the title and abstract, forty-five studies were selected for full-text evaluation. By full-text examination, twenty-five articles were eventually included for data synthesis with 2,027,512 participants and 103,734 CVD deaths [11,13,14,15,16,17,18,23,24,25,26,27,28,29,30,31,32,33,34,35,36,37,38,39,40]. 

The characteristics of the included studies are shown in Table 1 and Table 2. Among these articles, sixteen were from Europe and America, seven from Asia, one from Oceania and one from five continents. The range of the age was 18–84 years old. The population in the study included males and females. Besides, follow-up duration ranged from 5–30 years and the NOS quality score ranged from 6–9 points (Tables S1 and S2).

### 3.2. Fish Consumption and Cardiovascular Disease Mortality Risk

**Figure 2:**
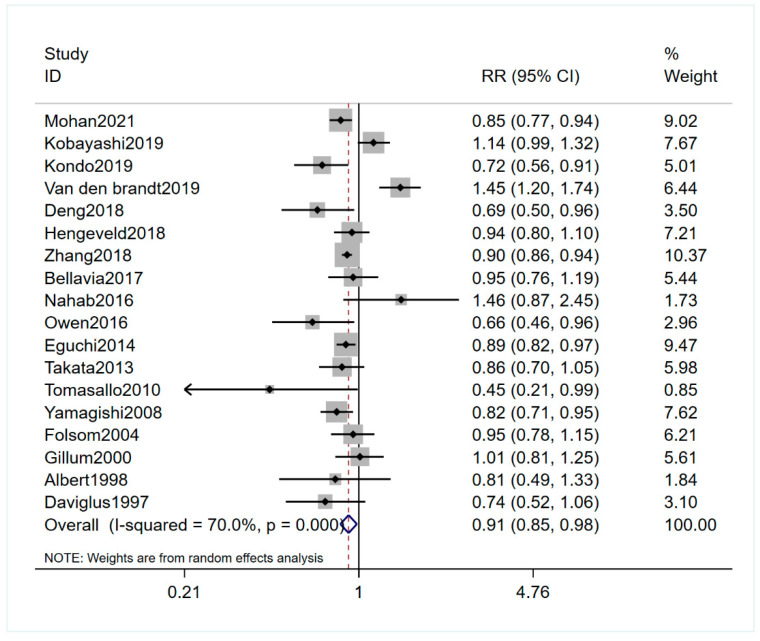
Forest plot of the highest versus lowest fish intake category and CVD mortality risk. Plot demonstrates decreased risk of CVD mortality risk with fish intake by the random-effects model (RR = 0.91; 95% CI, 0.85–0.98). CVD, cardiovascular disease; RR, relevant risk; CI, confidence intervals.

Eighteen studies, involving 1,267,951 participants and 51,628 CVD deaths, investigated the association between the fish intake and the CVD mortality risk [13,14,15,16,17,18,23,24,25,26,27,28,29,30,31,32,33,40]. The pooled RR (95% CI) was 0.91 (0.85–0.98) for the highest versus the lowest fish consumption category (I^2^ = 70.0%) (Figure 2). Sensitivity analysis did not change the protective effects of fish on CVD mortality (Figure S1). Subgroup analysis suggested that there was a significant negative association between the fish intake and the CVD mortality risk among the subgroups with nine years or more follow-up duration (Table 3). No publication bias was found (Egger’s test: *p* = 0.919; funnel plot: Figure S2).

**Figure 3:**
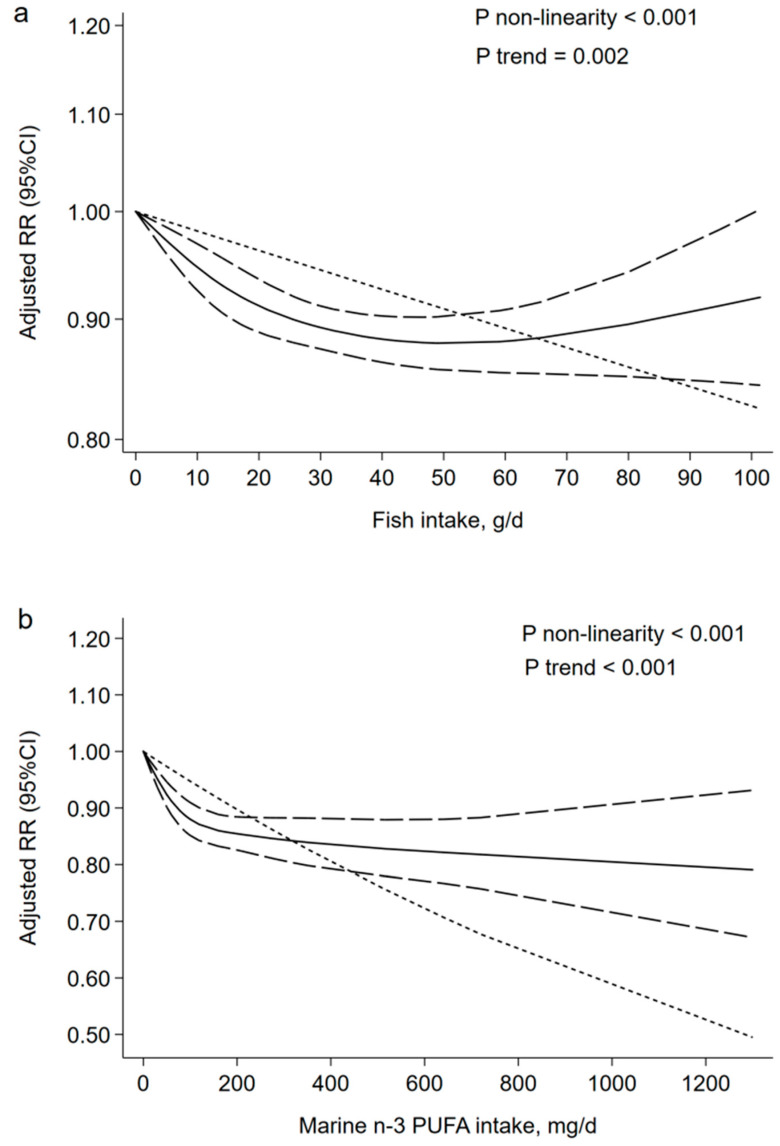
Dose-response association: (**a**) fish and CVD mortality (*n* = 10, *p* _non-linearity_ < 0.001; *p* _trend_ = 0.002); the risk of CVD mortality was decreased by 4% with an increase of 20 g of fish intake (RR = 0.96; 95% CI: 0.94–0.99) per day. (**b**) marine n-3 PUFA and CVD mortality (*n* = 8, *p* _non-linearity_ < 0.001; *p* _trend_ < 0.001); the risk of CVD mortality was decreased by 4% with an increase of 80 milligrams of marine n-3 PUFA intake (RR = 0.96; 95% CI: 0.94–0.98) per day. CVD, cardiovascular disease; n-3 PUFA, n-3 polyunsaturated fatty acids; RR, relevant risk; CI, confidence intervals; g/d, grams per day; mg/d, milligrams per day.

Figure 3a showed the linear and non-linear dose-response analyses between the fish intake and the CVD mortality risk. Ten prospective cohort studies met the requirements for dose-response analysis [13,15,16,17,18,23,27,29,33,40], and the curvilinear correlation presented a downward trend for the adjusted RR of CVD deaths with the increase of fish consumption from zero to 40 g/d (*p* _non-linearity_ < 0.001). The adjusted RR reached a steady value when fish consumption increased beyond 40 g/d. In the linear dose-response analysis, the summary RR (95% CI) for a 20 g/d increment was 0.96 (0.94–0.99) for CVD mortality risk (*p* _trend_ = 0.002).

### 3.3. Marine n-3 PUFA and Cardiovascular Disease Mortality Risk

**Figure 4:**
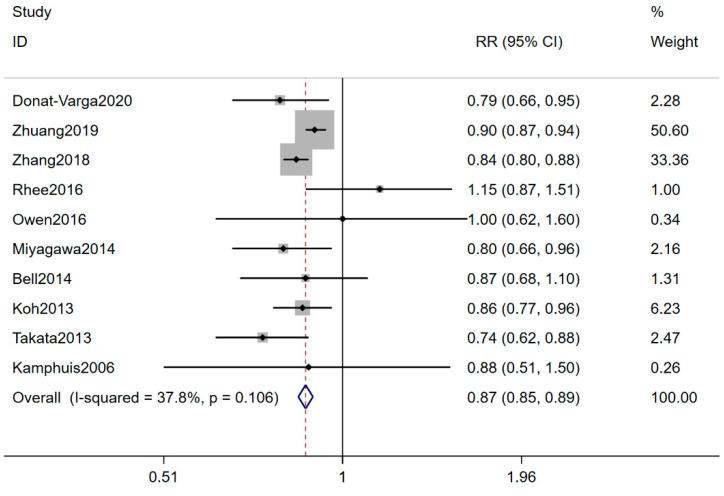
Forest plot of the highest versus lowest marine n-3 PUFA intake category and CVD mortality risk. Plot demonstrates decreased risk of CVD mortality risk with n-3 PUFA intake by the fixed-effects model (RR = 0.87; 95% CI, 0.85–0.89). CVD, cardiovascular disease; n-3 PUFA, n-3 polyunsaturated fatty acids; RR, relevant risk; CI, confidence intervals.

Ten eligible studies with 1,337,660 participants and 76,537 CVD deaths explored the association of marine n-3 PUFA intake with CVD mortality risk [11,18,25,27,34,35,36,37,38,39]. The pooled RR (95% CI) for the highest versus the lowest marine n-3 PUFA consumption category was 0.87 (0.85–0.89), with a low heterogeneity (I^2^ = 37.8%) (Figure 4). Sensitivity analysis suggested a great impact on one article with high quality (Figure S3) [35]. The negative association between marine n-3 PUFA and the risk of CVD mortality was altered from 0.87 (0.85–0.89) to 0.84 (0.81–0.87) by deleting this study. Subgroup analyses displayed a significant negative association among the Americas, and Asian and European countries compared with Oceania countries (Table 3). No publication bias was found (Egger’s test: *p* = 0.722; funnel plot: Figure S4). Figure 3b showed the linear and non-linear dose-response analysis between marine n-3 PUFA intake and CVD mortality risk. Eight prospective cohort studies met the requirements of dose-response analysis [18,25,27,34,36,37,38,39], and the curvilinear correlation presented a downward trend of CVD deaths with the increase of n-3 PUFA intake (*p* _non-linearity_ < 0.001). Linear dose-response analysis suggested that an increase of 80 milligrams of n-3 PUFA per day was associated with a 4% lower risk of CVD mortality (95% CI: 0.94–0.98; *p* _trend_ < 0.001).

## 4. Discussion

To our knowledge, the current work is the first meta-analysis of prospective observational studies for associating marine n-3 PUFA intake and CVD mortality risk. This study showed a significant inverse association between fish, marine n-3 PUFA intake and CVD mortality risk. Nonlinear dose-response relationship found that an increase of 20 g of fish intake or 80 milligrams of marine n-3 PUFA intake per day was associated with a 4% reduction in risk of CVD mortality.

In accordance with the previous study, the fish consumption was inversely associated with the CVD mortality risk in the current meta-analysis [12]. Bechthold et al.’s study also suggested a negative association between fish consumption and the risk of CVD [41]. Several studies showed no association between the fish intake and the risk of CVD [42,43]. Differences in preparation and type of fish might explain the observed difference. The progress of frying deteriorates oils through oxidation and hydrogenation, leading to an increase of trans fatty acids [44]. Trans fatty acids can aggravate inflammation and endothelial dysfunction, increasing the risk of CVD mortality [45]. Fish high in salt during cooking can increase the risk of CVD through increasing production of reactive oxygen species and oxidative stress, which contribute to impaired vascular function [46,47]. Fish can be divided into lean, medium-fatty or fatty fish with less than 2 g, 2–8 g and more than 8 g fat per 100 g in its body tissue [48]. Fatty fish diets significantly decreased the serum concentrations of triacylglycerol, apolipoprotein B, apolipoprotein CII and apolipoprotein CIII, which were known CVD risk markers [49]. Fishes also contain vitamin D, proteins, minerals and taurine which may decrease markers of inflammation and improve vascular function by increasing adiponectin levels [50]. In the subgroup of adjustment for diabetes, fish intake was associated with a reduction in the rate of major CVD mortality that approached significance (RR = 0.93; 95% CI: 0.85–1.01). Previous study has showed that supplementation of fish can decrease the CVD mortality risk in a diabetic population [51], the possible reason being that diabetes is a significant risk factor for CVD mortality [52]. EPA and DHA derived from fish can activate the G protein–coupled receptor 120 to reverse insulin resistance [53]. n-3 PUFA supplementation can protect against CVD in patients with diabetes [54]. 

In most studies where fish exits as an exposure variable, the observed benefits could often be attributed to the presence of fatty acids [55,56]. The long chain n-3 PUFA—namely, EPA and DHA—are naturally presented not only in fatty fish, but also in lean fish [57,58]. n-3 PUFA supplementation can decrease the risk of CVD [59,60]. The plasma level of EPA and DHA in humans may increase after intake of fish to improve the composition of lipoprotein cholesterol as cardiovascular markers affecting the risk of CVD [61,62]. However, previous study showed that low-dose supplementation with EPA and DHA did not significantly reduce the rate of CVD events [63]. This possible reason may be related to presence or absence of a history of CVD. The patients in the trial were all myocardial infarction patients for 4 years before enrollment. 85% of the patients were receiving statins. Patients with CVD who are receiving good clinical treatment showed low risk of future cardiovascular events [64]. Therefore, we wanted to observe the effect of the long chain n-3 PUFA on CVD mortality through the long-term duration.

In this meta-analysis, we also found a negative association between the marine n-3 PUFA intake and the CVD mortality risk. In previous studies, the results were not consistent [65]. A randomized controlled trial (RCT) showed that n-3 PUFA supplementation (866 mg/d) for 3.5 years could reduce CVD mortality risk [66]. In contrast, the RCT with one-year n-3 PUFA supplementation (850 mg/d) suggested no association [67]. Although some randomized controlled trials (RCTs) had been published, the follow-up duration were short with most studies ranged from 1–5 years [66,67,68]. Hoverer, the cohort studies included in this meta-analysis have longer follow-up duration ranged from 5–29 years. CVD is a chronic disease with a long disease course. Longer follow-up duration was more in line with the nature of the CVD disease. The possible mechanisms were as follows. First, the plasma n-3 PUFA increased with the frequency and the amount of dietary n-3 PUFA intake [69,70]. A higher circulating n-3 PUFA may alter the cell membrane fluidity which modulates protein function and signaling. The dimerization and recruitment of toll-like receptor-4 may be disrupted to down-regulate the expression of nuclear factor-kappaB reducing the inflammatory responses, with the enrichment of n-3 PUFA [71]. Second, n-3 PUFA may inhibit oxidative stress through the nuclear factor E2-related factor 2/heme oxygenase-1 signaling pathway. 4-hydroxy-2E-hexenal, the product of n-3 PUFA peroxidation, will dissociate Nrf2 from Keap1 and react with the cysteine residues of Keap1 [72]. Then, Nrf2 can translocate into the nucleus and bind to antioxidant responsive element to increase the expression of HO-1 [73]. HO-1 is a representative antioxidant enzyme that can confer cytoprotection on a wide variety of cells against oxidative damage [72]. Third, n-3 PUFA may reduce the hepatic very low-density lipoprotein production rate to decrease the plasma triglyceride levels through affecting fatty acid desaturases, fatty acid elongases and peroxisomal β- gene expression and fatty acid beta-oxidation [74,75]. In addition, long-chain n-3 PUFA may play an important role in improving the endothelial function, lowering circulating markers of endothelial dysfunction, such as E-selectin, vascular cell adhesion molecule-1 and intercellular adhesion molecule-1 [76,77,78].

The dose–response analysis showed that the risk of CVD mortality decreased with the increase of fish consumption from zero to 40 g/d. The adjusted RR reached a steady value when fish consumption increased beyond 40 g/d. Therefore, we believe that 40 g/d is the ideal dose for preventing CVD mortality. This is basically consistent with the average fish intake of the population of Europe and America [23,30]. However, the average intake of people in Japan is higher than this level [13].

This study has several strengths. First, compared with the previous meta-analysis [12], this study included additional 11 studies to investigate the association between the fish consumption and the CVD mortality risk, which may have a higher statistical power. Second, this meta-analysis was first to investigate the association between marine n-3 PUFA intake and CVD mortality risk with prospective cohort studies. Third, most studies had a long follow-up duration (9–30 years). CVD is a chronic disease and longer follow-up duration can better explain the association between fish, marine n-3 PUFA and CVD mortality risk.

The limitations should be acknowledged. First, several deep-sea fishes may be contaminated, while only one article reported whether fishes had pollutants or not [28]. Second, it is hard to standardize the fish and marine n-3 PUFA consumption due to the details of measurement methods not being available. Thus, we chose RR (95% CI) of the highest versus lowest fish and marine n-3 PUFA intake category and CVD mortality risk.

## 5. Conclusions

This meta-analysis indicated that the fish and marine n-3 PUFA intake were inversely associated with reduced risk of CVD mortality. This finding has important public health implications in terms of the prevention of CVD mortality. Since the biomarkers of fish and n-3 PUFA within an individual are important for food absorption, further research needs to be performed in biomarkers.

## Figures and Tables

**Table 1:** General characteristics of the studies included in meta-analysis of fish intake and risk of CVD mortality.

Author Name, Year, Country	Age Range/Mean Age (y)	Follow-Up Duration	Number of Cases/Size	Gender	Quantile	Adjusted RR (95% CI)	Quality Score	Adjustments
Mohan 2021, Asia, Africa, America, Europe and Oceania [40]	54.1	7.5	6502/191,454	M/F	4	0.85 (0.77–0.94)	6	Age, sex, study center, BMI, educational level, smoking status, alcohol intake, physical activity, urban or rural location, history of diabetes, cancer, use of statin or antihypertension medications, and intake of fruit, vegetables, red meat, poultry, dairy, and total energy
Kobayashi 2019, Japan [13]	45–74	14.9	2942/79,904	M/F	5	1.14 (0.99–1.32)	9	Age, area, BMI, alcohol intake total energy intake, coffee intake, green tea intake, smoking status, physical activity, occupation type, solitude and other food group
Kondo 2019, Japan [15]	30–79	29	1070/9115	M/F	3	0.72 (0.57–0.91)	8	Age, sex, smoking status, drinking status, and total energy intake
Van den brandt 2019, The Netherlands [16]	55–69	10	2985/120,852	M/F	4	1.45 (1.20–1.74)	9	Age at baseline, sex, cigarette smoking status, number of cigarettes smoked per day, years of smoking, diabetes, body height, non-occupational physical activity, highest level of education, intake of alcohol, vegetables and fruit, use of nutritional supplements and, in women, postmenopausal HRT
Deng 2018, USA [14]	≥18	18	326/1136	M/F	3	0.69 (0.50–0.96)	7	Age, sex, race/ethnicity, family income, the type of residential area, cigarette smoking, alcohol drinking, and the history of cardiovascular disease assessed at the baseline survey, and the years of using insulin as the indicator of diabetes severity
Hengeveld 2018, The Netherlands [17]	20–70	18	540/34,033	M/F	3	0.94 (0.80–1.10)	9	Age, sex, physical activity, smoking status, education level, BMI, alcohol intake, total energy intake, intakes of saturated fatty acids, trans fatty acids, fruit, vegetables, and dietary fiber
Zhang 2018, USA [18]	50–71	16	14824/240,729	M/F	5	0.9 (0.86–0.94)	8	Age, BMI, race, education, marital status, smoking, alcohol, intake of total energy, red meat, saturated fat, vegetables and fruits, multi-vitamin use, aspirin use, history of diabetes, history of hypertension, history of high cholesterol level
Bellavia 2017, Sweden [23]	45–83	17	5039/72,522	M/F	5	0.95 (0.94–0.95)	9	BMI, total physical activity, smoking status and pack-years of smoking, alcohol consumption, educational level (primary school, secondary school or university), total energy intake, fruit consumption, vegetable consumption, processed red meat consumption and non-processed red meat consumption
Nahab 2016, USA [24]	≥40	5.1	582/16,479	M/F	4	1.46 (0.87–2.45)	7	Age, race, region, sex, income, education, exercise, smoking status, Mediterranean diet score, regular aspirin use, total energy intake (kcald^−1^), current use of hypertensive medication, diabetes status, systolic blood pressure, BMI and dyslipidaemia
Owen 2016, Australia [25]	≥25	9.7	277/11,247	M/F	4	0.66 (0.46–0.96)	7	Age, previous CVD, education, exercise, diabetes, total dietary energy and smoking
Eguchi 2014, Japan [26]	40–79	19.3	2412/42,946	M/F	2	0.89 (0.82–0.97)	8	Age, body mass index, history of hypertension, history of diabetes, education level, regular employment, perceived mental stress, and 7 health behaviors
Takata 2013, China [27]	40–74	8.7	1789/134,296	M/F	5	0.86 (0.70–1.05)	6	Age at baseline, total energy intake, income, occupation, education, comorbidity index, physical activity level, red meat intake, poultry intake, total vegetable intake, total fruit intake, smoking history, and alcohol consumption
Tomasallo 2010, USA [28]	45.8	12	44/1367	M/F	3	0.45 (0.21–0.99)	7	Age, sex, body mass index, and income at study baseline
Yamagishi 2008, Japan [29]	40–79	12.7	2045/57,972	M/F	5	0.82 (0.71–0.95)	7	Age, gender, history of hypertension and diabetes mellitus, smoking status, alcohol consumption, body mass index, mental stress, walking, sports, education levels, total energy, and dietary intakes of cholesterol, saturated and n-6 polyunsaturated fatty acids, vegetables, and fruit
Folsom 2004, USA [30]	55–69	14	1589/41,836	F	5	0.95 (0.78–1.15)	7	Age, energy intake, educational level, physical activity level, alcohol consumption, smoking status, pack-years of cigarette smoking, age at first livebirth, estrogen use, vitamin use, body mass index, waist/hip ratio, diabetes, hypertension, intake of whole grains, fruit and vegetables, red meat, cholesterol, and saturated fat
Gillum 2000, USA [31]	25–74	18.8	--/8825	M/F	4	1.01 (0.81–1.25)	9	Age, smoking, history of diabetes, education, high school graduate, systolic blood pressure, serum cholesterol concentration, body mass index, alcohol intake, and physical activity
Albert 1998 [32]	40–84	11	548/20,551	M	5	0.81 (0.49–1.33)	8	Age, aspirin and beta carotene treatment assignment, evidence of cardiovascular disease, prior to 12-month questionnaire, body mass index, smoking status, history of diabetes, history of hypertension, history of hypercholesterolemia, alcohol consumption, vigorous exercise, and vitamin E, vitamin C, and multivitamin use
Daviglus 1997 [33]	40–55	30	573/2107	M	4	0.74 (0.52–1.06)	8	Age, education, religion, systolic pressure, serum cholesterol, number of cigarettes smoked per day, body-mass index, presence or absence of diabetes, presence or absence of electrocardiographic abnormalities, daily intake of energy, cholesterol, saturated, monounsaturated, and polyunsaturated fatty acids, total protein, carbohydrate, alcohol, iron, thiamine, riboflavin, niacin, vitamin C, beta carotene, and retinol

CVD, cardiovascular disease; BMI, body mass index; HRT, hormone replacement therapy.

**Table 2:** General characteristics of the studies included in meta-analysis of marine n-3 PUFA intake and risk of CVD mortality.

Author Name, Year, Country	Age Range/Mean Age (y)	Follow-Up Duration	Number of Cases/Size	Gender	Quantile	Adjusted RR (95% CI)	Quality Score	Adjustments
Donat-Varga 2020, Sweden [34]	Men: 65.5 Women: 62.5	15.5	6338/69,497	M/F	5	0.79 (0.66–0.95)	8	Age, gender, education level, waist circumference, hypertension, hypercholesterolemia, weight loss > 5kg within 1 year, leisure-time inactivity and daily walking/cycling, family history of myocardial infarction before the age of 60 years, smoking status, use of aspirin, energy intake, Mediterranean diet, parity, use of hormone replacement therapy and dietary methylmercury exposure, dietary PCB exposure
Zhuang 2019, USA [35]	50–71	16	38,747/521,120	M/F	5	0.9 (0.87–0.94)	8	Age, gender, BMI, race, education, marital status, household income, smoking, alcohol drinking, physical activity, multi-vitamin use, aspirin use, history of hypertension, history of hypercholesterolemia, perceived health condition, history of heart disease, stroke, diabetes, and cancer at baseline, hormones use for women, intake of total energy, percentages of energy intake from protein, and remaining fatty acids where appropriate (saturated, α-linolenic, marine omega-3, linoleic, arachidonic, monounsaturated and trans fatty acids)
Zhang 2018, USA [18]	50–71	16	22,365/421,309	M/F	5	0.84 (0.80–0.88)	8	Age, BMI, race, education, marital status, smoking, alcohol, intake of total energy, red meat, saturated fat, vegetables and fruits, physical activity, multi-vitamin use, aspirin use, history of diabetes, history of hypertension, history of high cholesterol level and hormones use, intake of a-linolenic acid, omega-6 PUFAs, monounsaturated fatty acids and trans-fatty acid
Rhee 2016, USA [11]	≥45	22	501/39,876	F	5	1.15 (0.87–1.51)	9	Age, BMI, smoking, alcohol intake, physical activity, randomized treatment, oral contraceptive use, use of hormones as defined under HRT, multivitamin use, family history of MI, baseline history of hypertension, high cholesterol, and diabetes, intakes of dietary fiber, fruits and vegetables, trans fat, ratio of polyunsaturated to saturated fat, and sodium
Owen 2016, Australia [25]	≥25	9.7	277/11,247	M/F	5	1.00 (0.62–1.60)	7	Age, sex, previous CVD, education, exercise, diabetes, total dietary energy and smoking
Miyagawa 2014, Japan [36]	≥30	24	879/9190	M/F	4	0.80 (0.66–0.96)	7	Age, sex, smoking status, drinking status, systolic blood pressure, blood glucose, serum total cholesterol, body mass index, antihypertensive medication status, residential area, dietary intakes of saturated fatty acids, total n-6 PUFA, vegetable protein, total dietary fiber and sodium
Bell 2014, USA [37]	50–76	5	769/70,495	M/F	4	0.87 (0.68–1.10)	6	Age, sex, raceethnicity, marital status, education, body mass index, physical activity, smoking, alcohol intake, total energy intake, vegetables intake, dietary intake of arachidonic acid, aspirin use, use of non-aspirin nonsteroidal anti-inflammatory drugs, self-rated health, sigmoidoscopy, mammogram, prostate-specific antigen test, current use of cholesterol-lowering medication, history of cardiovascular disease, family history of heart attack, current use of blood pressure medication, percentage of calories derived from trans-fat, percentage of calories derived from saturated fat, years of estrogen therapy, and years of estrogen + progestin therapy etc.
Koh 2013, Singapore [38]	45–74	14.8	4780/60,298	M/F	4	0.86 (0.77–0.96)	8	Age, sex, dialect, year of interview, educational level, body mass index, physical activity, smoking status, alcohol use, baseline history of self-reported diabetes, hypertension, coronary heart disease, stroke, and total energy, adjusted for intakes of protein, dietary fiber, monounsaturated fat, saturated fat, omega-6 fatty acids, and alternate omega-3 fatty acids
Takata 2013, China [27]	40–74	8.7	1789/134,296	M/F	5	0.74 (0.62–0.88)	6	Age, total energy intake, income, occupation, education, comorbidity index, physical activity level, red meat intake, poultry intake, total vegetable intake, total fruit intake, smoking history, and alcohol consumption (among men only)
Kamphuis 2006, The Netherlands [39]	70–79	10	92/332	M	3	0.88 (0.51–1.5)	8	Age, years of education, BMI, smoking, alcohol consumption, systolic blood pressure, total and HDL-cholesterol concentrations, physical activity, living alone, and energy intake

n-3 PUFA, n-3 polyunsaturated fatty acid; CVD, cardiovascular disease; PCB, polychlorinated biphenyl; BMI, body mass index; HRT, hormone replacement therapy; MI, myocardial infarction.

**Table 3:** Subgroup and meta-regression analyses for the association between fish, n-3 PUFA intake and CVD mortality.

Comparison		N ^†^	Pooled RRs (95% CI)	Heterogeneity (I^2^), *p* ^a^ Value	*p*^b^ Value	*p*^c^ Value
Fish Intake and CVD Mortality Risk		18	0.91 (0.85–0.98)	70.0%, 0.000	0.015	
Country	Asia	5	0.89 (0.78–1.01)	74.1%, 0.004	0.081	0.216
	Europe and America	11	0.95 (0.84–1.08)	72.2%, 0.000	0.417	
	Oceania	1	0.66 (0.46–0.95)	--	0.027	
	Asia, Africa, America, Europe and Oceania	1	0.85 (0.77, 0.94)	--	0.001	
Gender	Men	2	0.76 (0.57–1.02)	0.0%, 0.773	0.067	0.442
	women	1	0.95 (0.78–1.15)	--	0.605	
	Both	15	0.92 (0.85–1.00)	74.5%, 0.000	0.040	
Follow-up duration	<9 years	3	0.90 (0.76–1.07)	50.6%, 0.132	0.234	0.851
	≥9 years	15	0.91 (0.84–0.99)	72.7%, 0.000	0.035	
Dropout rate	<20%	11	0.93 (0.82–1.06)	76.7%, 0.000	0.284	0.557
	>20%	7	0.88 (0.82–0.94)	41.6%, 0.113	0.000	
Excluding history of CVD	Yes	11	0.97 (0.88–1.06)	77.0%, 0.000	0.492	0.905
	No	7	0.82 (0.75–0.91)	21.7%, 0.264	0.000	
Adjustment for diabetes	Yes	11	0.93 (0.85, 1.01)	72.9%, 0.000	0.094	0.040
	No	4	0.84 (0.63, 1.12)	80.9%, 0.001	0.233	
	Others *	3	0.89 (0.76, 1.04)	34.5%, 0.217	0.149	
Adjustment for smoking	Yes	16	0.92 (0.85, 1.00)	71.8%, 0.000	0.050	0.484
	No	2	0.71 (0.38, 1.33)	66.0%, 0.087	0.285	
Marine n-3 PUFA and CVD mortality risk		10	0.87 (0.85–0.89)	37.8%, 0.106	0.000	
Country	Asia	3	0.82 (0.75–0.89)	4.9%, 0.349	0.000	0.212
	Europe and America	6	0.88 (0.85–0.90)	49.2%, 0.08	0.000	
	Oceania	1	1.00 (0.62–1.61)	--	1.000	
Gender	Men	1	0.88 (0.51–1.51)	--	0.642	0.182
	Women	1	1.15 (0.87–1.52)	--	0.320	
	Both	8	0.87 (0.84–0.89)	33.3%, 0.162	0.000	
Follow-up duration	<9 years	2	0.78 (0.68-0.90)	12.1%, 0.286	0.001	0.192
	≥9 years	8	0.87 (0.85–0.90)	37.0%, 0.134	0.000	
Dropout rate	<20%	5	0.89 (0.86–0.92)	51.8%, 0.08	0.000	0.114
	>20%	5	0.84 (0.80–0.87)	0.0%, 0.877	0.000	
Excluding history of CVD	Yes	5	0.84 (0.81–0.88)	29.9%, 0.222	0.000	0.536
	No	5	0.89 (0.86–0.92)	23.4%, 0.266	0.000	
Adjustment for diabetes	Yes	6	0.88 (0.85, 0.90)	44.4%, 0.109	0.000	0.060
	No	3	0.77 (0.68, 0.88)	0.0%, 0.745	0.000	
	Others *	1	0.79 (0.66, 0.95)	--	0.0 11	

N ^†^ Number of included studies; *p* ^a^ for heterogeneity; *p* ^b^ for significance test; *p* ^c^ for meta-regression analysis. Others * All patients were diabetic or not diabetic. n-3 PUFA, n-3 polyunsaturated fatty acid; CVD, cardiovascular disease.

## Supplementary Materials

The following are available online at https://www.mdpi.com/article/10.3390/nu13072342/s1, Figure S1: Sensitivity analysis with respect to fish intake and CVD mortality risk. Figure S2: Funnel plot of the RR of 18 articles on fish intake and CVD mortality risk. Figure S3: Sensitivity analysis with respect to marine n-3 PUFA intake and CVD mortality risk. Figure S4: Funnel plot of the RR of 10 articles on marine n-3 PUFA intake and CVD mortality risk. Table S1: Quality assessment of studies investigating fish intake and CVD mortality risk. Table S2: Quality assessment of studies investigating marine n-3 PUFA intake and CVD mortality risk.
