# Supplementary material for: Intake of Fish and Marine n-3 Polyunsaturated Fatty Acids and Risk of Cardiovascular Disease Mortality: A Meta-Analysis of Prospective Cohort Studies
Source: Nutrients. 2021 Jul 9;13(7):2342. doi: 10.3390/nu13072342 (PMC8308510; doi:10.3390/nu13072342)
Supplement: Supplementary file 1 [file nutrients-13-02342-s001.zip › nutrients-1250006-SI.pdf]

Supporting Information

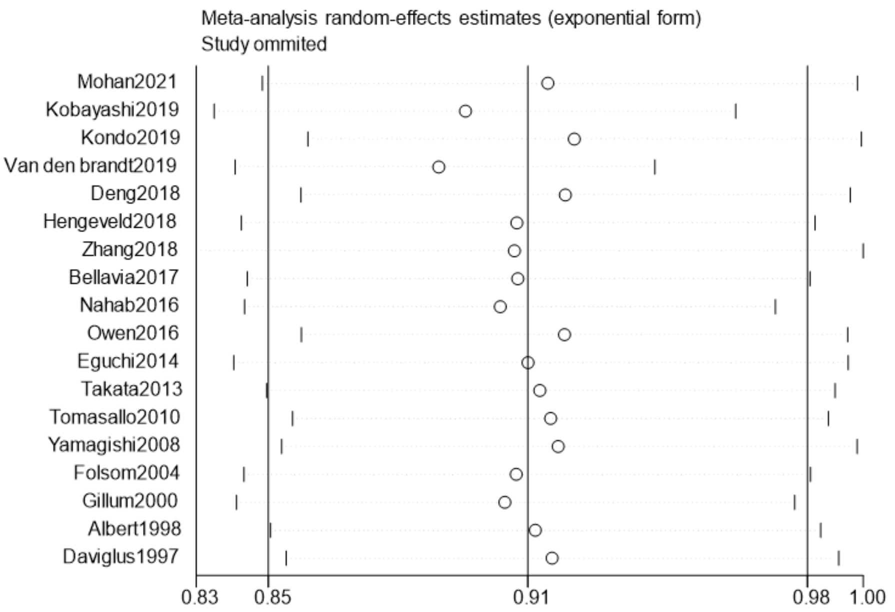

Figure S1. Sensitivity analysis with respect to fish intake and CVD mortality risk. Sensitivity analysis did not change the results. CVD, cardiovascular disease.

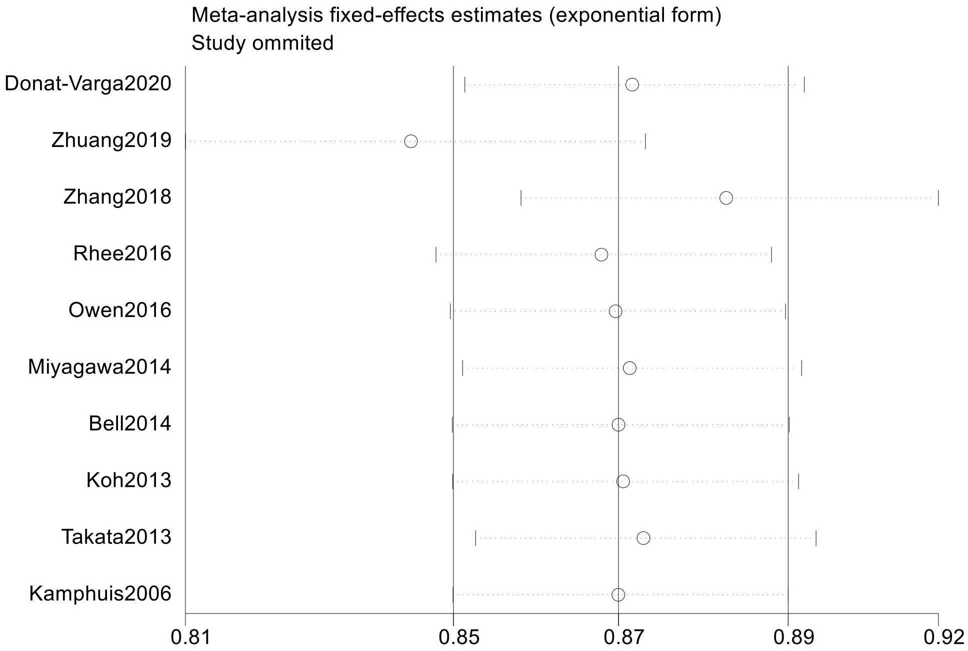

Figure S2. Sensitivity analysis with respect to marine n-3 PUFA intake and CVD mortality risk. The negative association between marine n-3 PUFA and risk of CVD mortality was altered from 0.87 (0.85-0.89) to 0.84 (0.81-0.87) by deleting the study of Zhuang2019. n-3 PUFA, n-3 polyunsaturated fatty acids; CVD, cardiovascular disease.

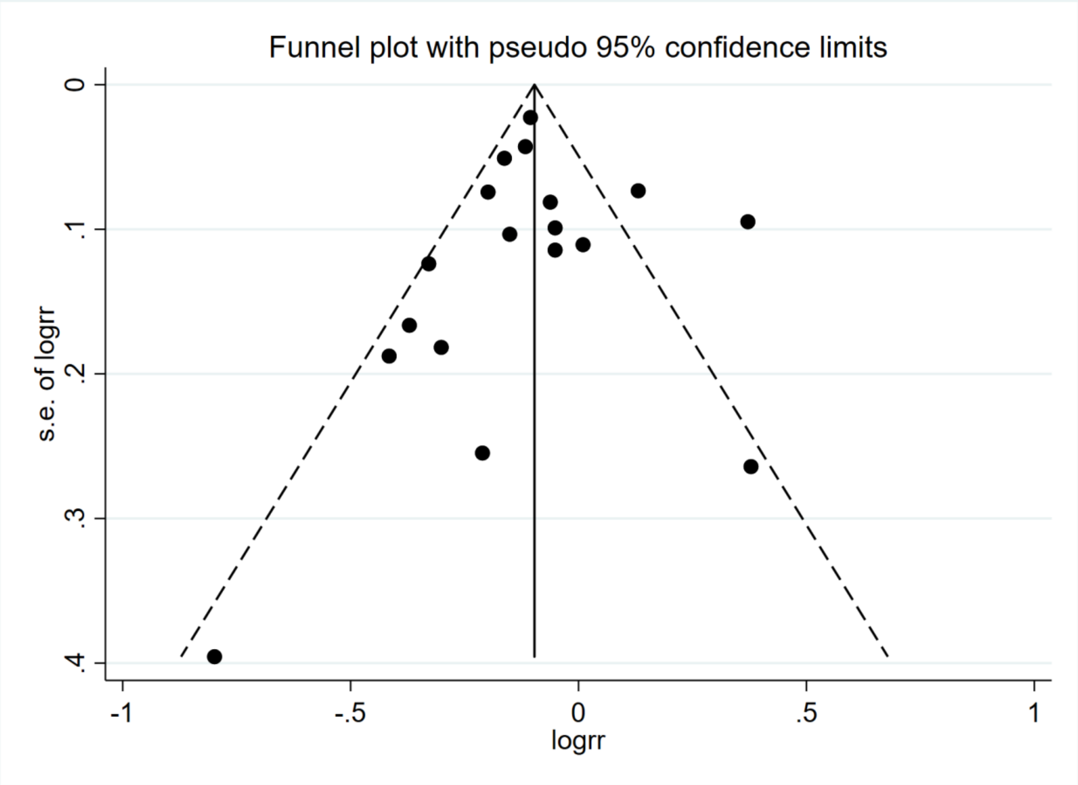

Figure S3. Funnel plot of the RR of 18 articles on fish intake and CVD mortality risk. No evidence of publication bias was noted. RR, relative risk; CVD, cardiovascular disease.

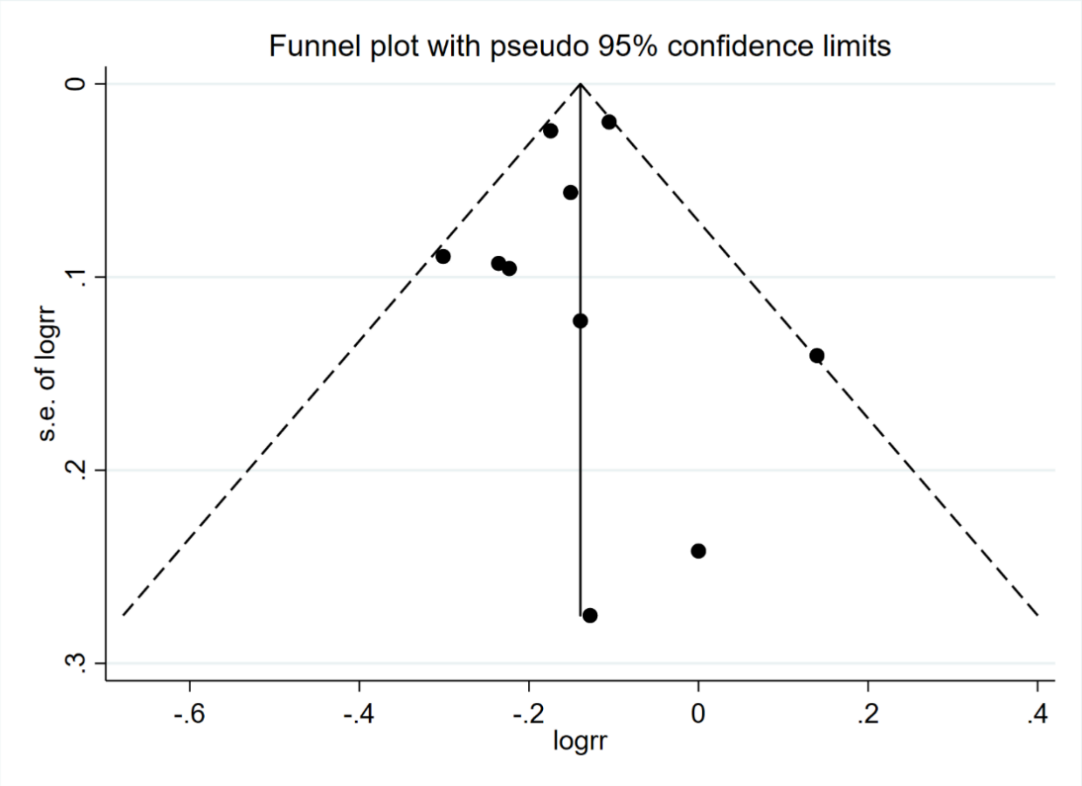

Figure S4. Funnel plot of the RR of 10 articles on marine n-3 PUFA intake and CVD mortality risk. No evidence of publication bias was noted. RR, relative risk; n-3 PUFA, n-3 polyunsaturated fatty acids; CVD, cardiovascular disease.

Table S1. Quality assessment of studies investigating fish intake and CVD mortality risk.

Study	Representativeness of the Exposed Cohort	Selection of the Non exposed Cohort	Ascertainment of Exposure	Demonstration that Outcome of Interest at Start of Study	Comparability of Cohorts on the Basis of the Design or Analysis	Assessment of outcome	Follow-Up Long Enough for the Outcomes to Occur	Adequacy of Follow-Up of Cohorts	Grades
Mohan 2021	-	☆	☆	-	☆☆	☆	-	☆	☆☆☆☆☆☆
Kobayashi2019	☆	☆	☆	☆	☆☆	☆	☆	☆	☆☆☆☆☆☆☆☆☆
Kondo 2019	☆	☆	☆	☆	☆	☆	☆	☆	☆☆☆☆☆☆☆☆
Van den brandt 2019	☆	☆	☆	☆	☆☆	☆	☆	☆	☆☆☆☆☆☆☆☆☆
Deng2018	☆	☆	☆	-	☆☆	☆	☆	-	☆☆☆☆☆☆☆
Henge	☆	☆	☆	☆	☆☆	☆	☆	☆	☆☆☆☆

veld2018									☆☆☆☆☆
Zhang 2018	☆	☆	☆	☆	☆☆	☆	☆	-	☆☆☆☆☆☆☆☆
Bellavia2017	☆	☆	☆	☆	☆☆	☆	☆	☆	☆☆☆☆☆☆☆☆☆
Nahab 2016	☆	☆	☆	☆	☆☆	☆	-	-	☆☆☆☆☆☆☆
Owen2016	☆	☆	☆	-	☆☆	☆	-	☆	☆☆☆☆☆☆☆
Eguchi 2014	☆	☆	☆	☆	☆☆	☆	☆	-	☆☆☆☆☆☆☆☆
Takata 2013	☆	☆	☆	-	☆	☆	-	☆	☆☆☆☆☆☆
Tomasallo2010	☆	☆	☆	-	☆	☆	☆	☆	☆☆☆☆☆☆☆
Yamag	☆	☆	☆	☆	☆	☆	☆	-	☆☆☆☆

ishi2008									☆☆☆
Folsom2004	☆	☆	☆	-	☆☆	☆	☆	-	☆☆☆☆☆☆☆
Gillum 2000	☆	☆	☆	☆	☆☆	☆	☆	☆	☆☆☆☆☆☆☆☆☆
Albert 1998	-	☆	☆	☆	☆☆	☆	☆	☆	☆☆☆☆☆☆☆☆
Daviglus1997	☆	☆	☆	-	☆☆	☆	☆	☆	☆☆☆☆☆☆☆☆

CVD, cardiovascular disease

Table S2. Quality assessment of studies investigating marine n-3 PUFA intake and CVD mortality risk.

Study	Representativeness of the Exposed Cohort	Selection of the Non exposed Cohort	Ascertainment of Exposure	Demonstration that Outcome of Interest at Start of Study	Comparability of Cohorts on the Basis of the Design or Analysis	Assessment of outcome	Follow-Up Long Enough for the Outcomes to Occur	Adequacy of Follow-Up of Cohorts	Grades
Donat-Varga2020	☆	☆	☆	☆	☆☆	☆	☆	-	☆☆☆☆☆☆☆☆
Zhuang2019	☆	☆	☆	-	☆☆	☆	☆	☆	☆☆☆☆☆☆☆☆
Zhang2018	☆	☆	☆	☆	☆☆	☆	☆	-	☆☆☆☆☆☆☆☆
Rhee2016	☆	☆	☆	☆	☆☆	☆	☆	☆	☆☆☆☆☆☆☆☆☆
Owen2016	☆	☆	☆	-	☆☆	☆	-	☆	☆☆☆☆☆☆☆
Miyagawa2014	☆	☆	☆	☆	☆	☆	☆	-	☆☆☆☆☆☆☆
Bell2014	☆	☆	☆	-	☆	☆	-	☆	☆☆☆☆☆☆
Koh2013	☆	☆	☆	-	☆☆	☆	☆	☆	☆☆☆☆☆☆☆☆
Takata2013	☆	☆	☆	-	☆	☆	-	☆	☆☆☆☆☆☆
Kamphuis2006	☆	☆	☆	☆	☆☆	☆	☆	-	☆☆☆☆☆☆☆☆

CVD, cardiovascular disease
